# Transfemoral trans-facial vein-superior ophthalmic vein to embolize cavernous sinus dural arteriovenous fistulas

**DOI:** 10.3389/fneur.2022.1078185

**Published:** 2023-01-12

**Authors:** Zibo Zhou, Kan Xu, Jinlu Yu

**Affiliations:** Department of Neurosurgery, First Hospital of Jilin University, Changchun, China

**Keywords:** facial vein, cavernous sinus, dural arteriovenous fistula, embolization, review

## Abstract

Cavernous sinus dural arteriovenous fistula (CS-DAVF) is an abnormal communication between the CS and dural arteries from the internal carotid artery and external carotid artery. CS-DAVFs are not uncommon. The preferred treatment for most CS-DAVFs is transvenous embolization (TVE), which can achieve a high cure rate with few complications. The trans-inferior petrous sinus (IPS) route from the internal jugular vein to the CS is the favorite and most direct route to perform TVE in the great majority of CS-DAVFs. However, when the trans-IPS route fails and if the facial vein (FV) is patent and dilated, transfemoral trans-FV-superior ophthalmic vein (SOV) embolization of the CS-DAVF can be attempted. However, the transfemoral trans-FV-SOV route to embolize CS-DAVFs is often challenging, and there is insufficient knowledge about it. Therefore, an updated review of the transfemoral trans-FV-SOV route to embolize CS-DAVFs is necessary, and this review includes our experience. The images in this review are from our institute without the dispute of copyright. Issues regarding the transfemoral trans-FV-SOV route to embolize CS-DAV were discussed, including the FV anatomy and variation, various TVE routes to access CS-DAVF, the procedure of the transfemoral trans-FV-SOV route to embolize CS-DAVF, difficulty, and solution of the transfemoral trans-FV-SOV route to embolize CS-DAVF, and complications and prognosis of transfemoral trans-FV-SOV to embolize CS-DAVF. By reviewing the transfemoral trans-FV-SOV route to embolize CS-DAVFs, we found that this route provides a valuable alternative to the other transvenous routes. A good prognosis can be obtained with the transfemoral trans-FV-SOV route to embolize CS-DAVFs in select cases.

## 1. Introduction

Cavernous sinus dural arteriovenous fistula (CS-DAVF) is an abnormal communication between the CS and dural arteries from the internal carotid artery (ICA) and external carotid artery (ECA), and these fistulas are located in the dura within or near the CS wall (1). CS-DAVF is the second most common intracranial DAVF after transverse-sigmoid sinus DAVF and accounts for ~16% of intracranial DAVFs (1, 2). In the Asian literature, CS-DAVF is the most frequent fistula (3, 4). Low-flow CS-DAVFs may have a self-limiting behavior and can close spontaneously, but high-flow CS-DAVFs do not (5).

**Figure 1:**
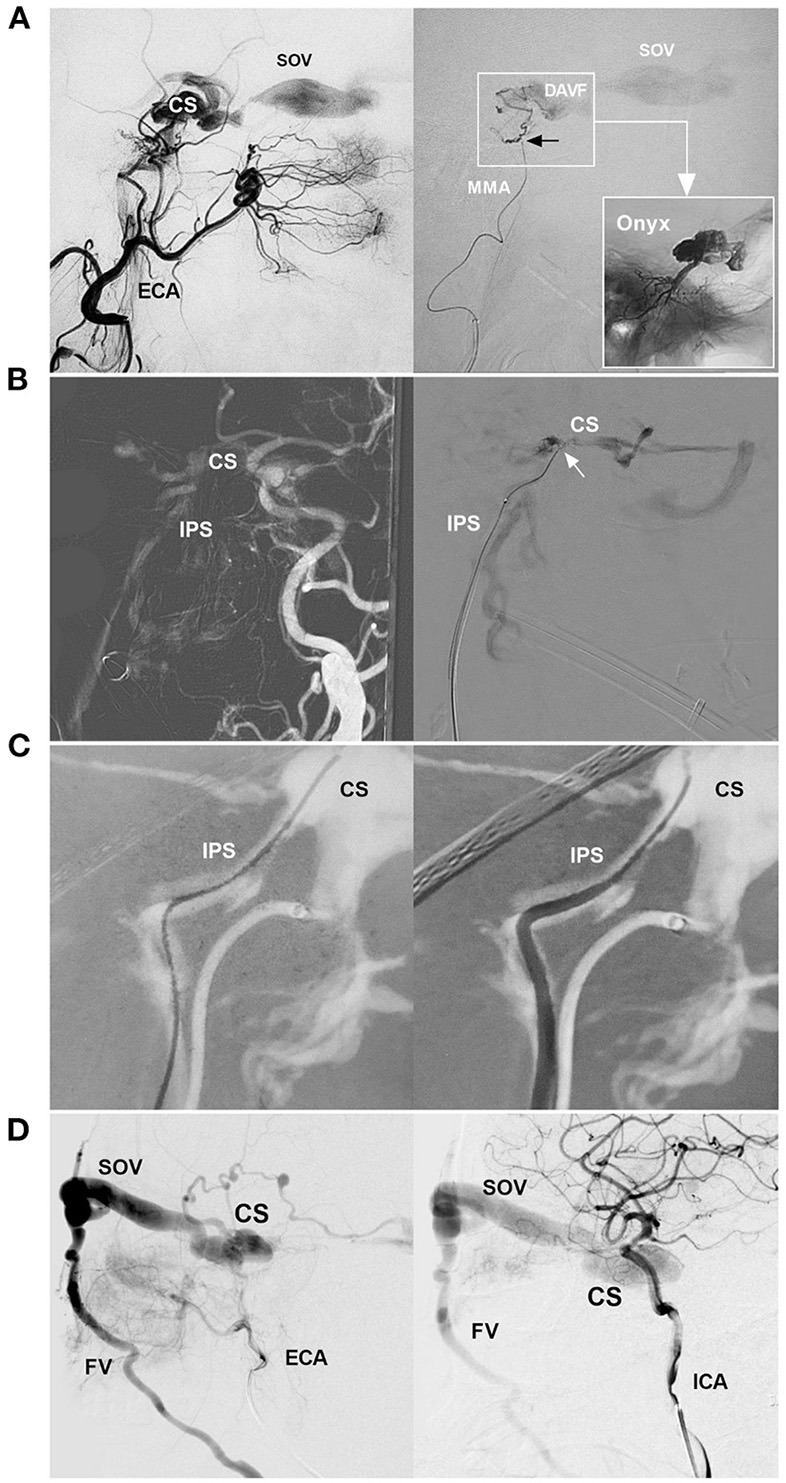
EVT routes of CS-DAVF. **(A)** EVT for CS-DAVF *via* the MMA. Left: angiography of the ECA shows the CS-DAVF draining into the SOV; Right: superselective angiography of the MMA confirmed that the microcatheter obtained a wedged position (black arrow) and accessed the fistula (frame); the picture in a picture (right arrow) shows the casting of Onyx in the CS. **(B)** EVT for CS-DAVF *via* IPS. Left: Navigation image of the road map shows that the CS-DAVF drained into the IPS; Right: Superselective angiography confirmed that the microcatheter (arrow) was positioned in the CS. **(C)** Catheterization *via* IPS. Navigation image of the road map shows the guidewire into the CS *via* the IPS (left); then, the catheter went into the IPS following the guidewire (right). **(D)** CS-DAVF with the SOV as the main draining vein. Angiographies of the ECA (left) and ICA (right) show a CS-DAVF drained *via* the SOV and then to the FV. The CS-DAVF was appropriated for the trans-FV-SOV route to perform EVT. CS, cavernous sinus; DAVF, dural arteriovenous fistula; ECA, external carotid artery; EVT, endovascular treatment; FV, facial vein; ICA, internal carotid artery; IPS, inferior petrous sinus; MMA, middle meningeal artery; SOV, superior ophthalmic vein.

Treatment is recommended for high-flow CS-DAVFs, especially those with visual symptoms or cortical venous drainage (6). Currently, endovascular treatment (EVT) represents the first-line therapy for CS-DAVF, and EVT includes transarterial embolization (TAE), transvenous embolization (TVE), or both (7). In CS-DAVFs, TAE is often difficult because most CS-DAVFs are fed by numerous and tiny dural branches from the ICA and ECA and can only be used in highly selected cases (Figure 1A).

In almost two-third of CS-DAVFs, TVE is the preferred treatment and can achieve a high percentage of radiological and clinical resolution with a low complication rate (Figures 1B, C) (1). The trans-inferior petrous sinus (IPS) route from the trans-internal jugular vein (IJV) to the CS is the favorite and most direct route to perform TVE (8). In TVEs, if the facial vein (FV) is patent, the trans-FV-superior ophthalmic vein (SOV) route *via* the transfemoral trans-IJV or trans-external jugular vein (EJV) can be attempted (Figure 1D) (9).

However, because of the considerable variation in the anatomy of the head and neck veins, the transfemoral trans-FV-SOV route to embolize CS-DAVF is challenging (10, 11). The understanding of the technique was insufficient. Therefore, an updated review of transfemoral trans-FV-SOV routes to embolize CS-DAVFs is necessary. In addition, we also provide educational images and cases to increase reading interest and show our experience. The images are from our institute without the dispute of copyright.

## 2. FV anatomy and variation

### 2.1. Typical anatomy

**Figure 2:**
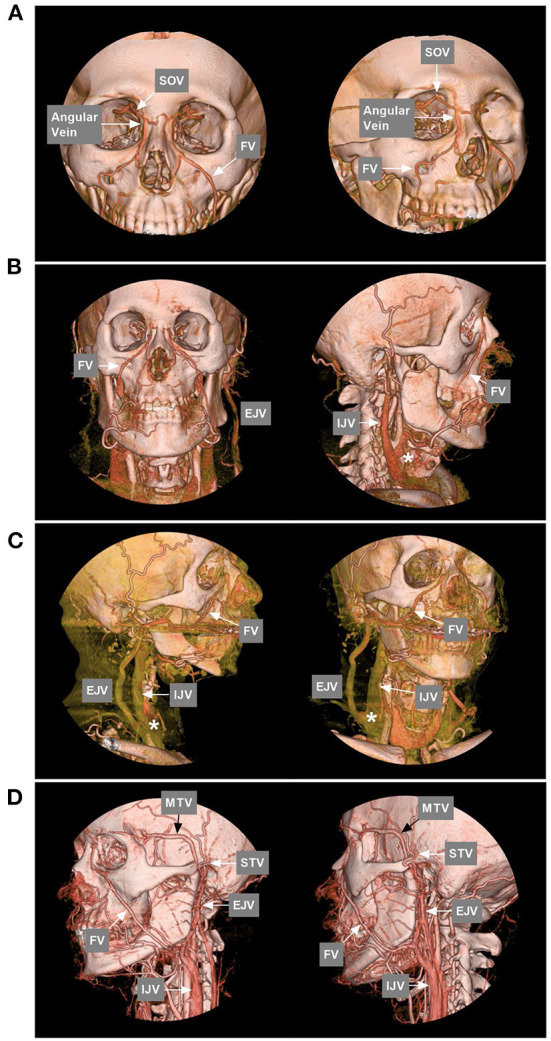
FV anatomy of CTA. **(A)** Anterior–posterior view (left) and oblique (right) view of CTA show that the SOV from the CS connected with the angular vein; then, the angular vein continued as the FV. **(B)** Anterior–posterior view (left) and oblique (right) view of CTA show that the typical FV descends obliquely in a straight line to continue the common FV and flow with the IJV. The asterisk (right) indicates the junction of the FV with the IJV. **(C)** Lateral view (left) and oblique (right) view of CTA show the FV together with the EJV into the IJV. The asterisks (left and right) indicate the junction of the FV into the IJV. **(D)** Lateral view (left) and oblique (right) view of CTA show that the MTV joins the STV to form the EJV and then connects with the FV into the IJV; in the face, many tidy veins join into the FV. CS, cavernous sinus; CTA, computed tomography angiography; EJV, external jugular vein; FV, facial vein; IJV, internal jugular vein; MTV, middle temporal vein; SOV, superior ophthalmic vein; STV, superficial temporal vein.

The FV (aka the anterior FV) originates from the angular vein at the nose root (12). Angular veins are formed by the confluence of the supratrochlear and supraorbital veins (13). The superior ophthalmic vein (SOV) and inferior ophthalmic veins that drain from the orbit connect to the angular vein, forming a communication between the FV and CS (Figure 2A) (14). These vessels form a complex orbital venous system with a variable network (15). The typical FV descends obliquely in a straight line and curves around the inferior edge of the mandible to merge with the submental and retromandibular veins to form the common FV and eventually flows into the IJV at different levels of the middle cervical region (Figures 2B, C) (16). Along its course, the FV has extensive connections with the medial temporal vein, superficial temporal vein, deep FV, and pterygoid plexus (Figure 2D).

### 2.2. Variation

**Figure 3:**
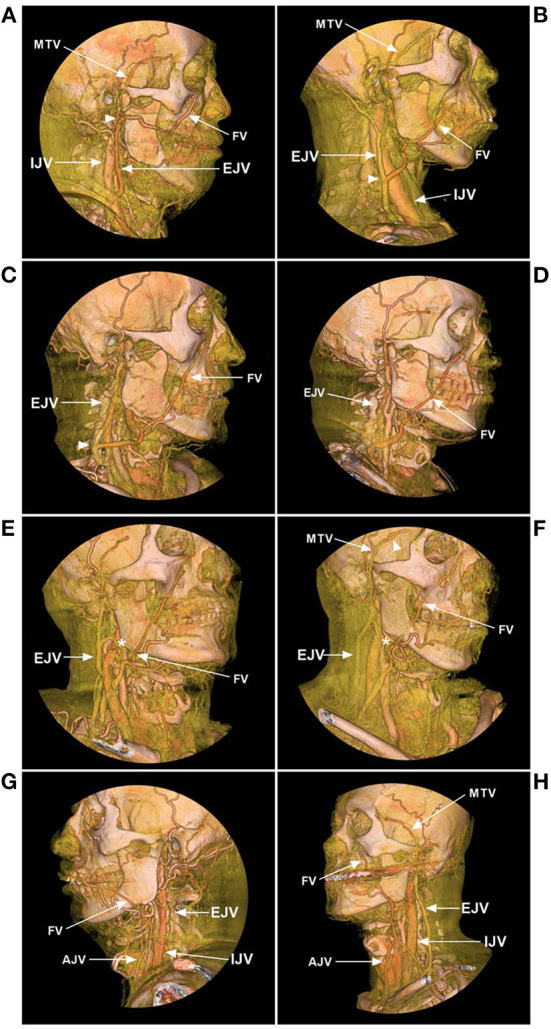
FV variance of CTA. **(A)** Lateral view CTA shows that the FV courses posteriorly and combines with the MTV at the mandibular joint (arrowhead) from the EJV. **(B)** Lateral view CTA shows that the FV descends obliquely and curves around the inferior edge of the mandible and flows into the EJV at the middle cervical region (arrowhead). **(C)** Lateral view CTA shows that the FV flows into the EJV in the low cervical region (arrowhead). **(D)** Lateral view CTA shows that the FV descends obliquely and curves around the inferior edge of the mandible and flows into the subclavian vein without connection with the IJV or EJV. **(E, F)** Oblique view CTAs shows that the FV flows into the subclavian vein with the connection with the EJV (asterisks). **(G, H)** Oblique view CTAs shows that the FVs mainly flow into the AJVs, with the connection with the EJVs. AJV, anterior jugular vein; CTA, computed tomography angiography; EJV, external jugular vein; FV, facial vein; IJV, internal jugular vein; MTV, middle temporal vein.

Except for the IJV, the FV can also drain into the EJV (Figures 3A–C) (17–19). Choudhry et al. (17) and Gupta et al. (18) reported that 5% and 9% of the FVs drain into the EVJ due to a persistent anastomotic channel between the primitive linguofacial vein and secondarily developed EVJ, respectively. In East Asia, the rate of FVs that drain into the EJV was higher than that in the earlier reports, which means that there are developmental venous variations in different races. For instance, in a Japanese report by Fujita et al. (20) that studied CS-DAVFs embolized with the transfemoral trans-FV-SOV route, 20% (2/10) of the patients had FVs that drained into the EJV. In an Asian Taiwan report by Luo et al. (21) that included 26 direct and indirect carotid-cavernous fistulas treated with the transfemoral trans-FV-SOV route, 62% of the patients were found to have FVs that drained into the EJV. In addition, the FV can directly drain into the subclavian vein with/without the connection of the IJV or EJV (Figures 3D–F). Rarely, the FV can directly drain into the anterior jugular vein (Figures 3G, H).

**Figure 4:**
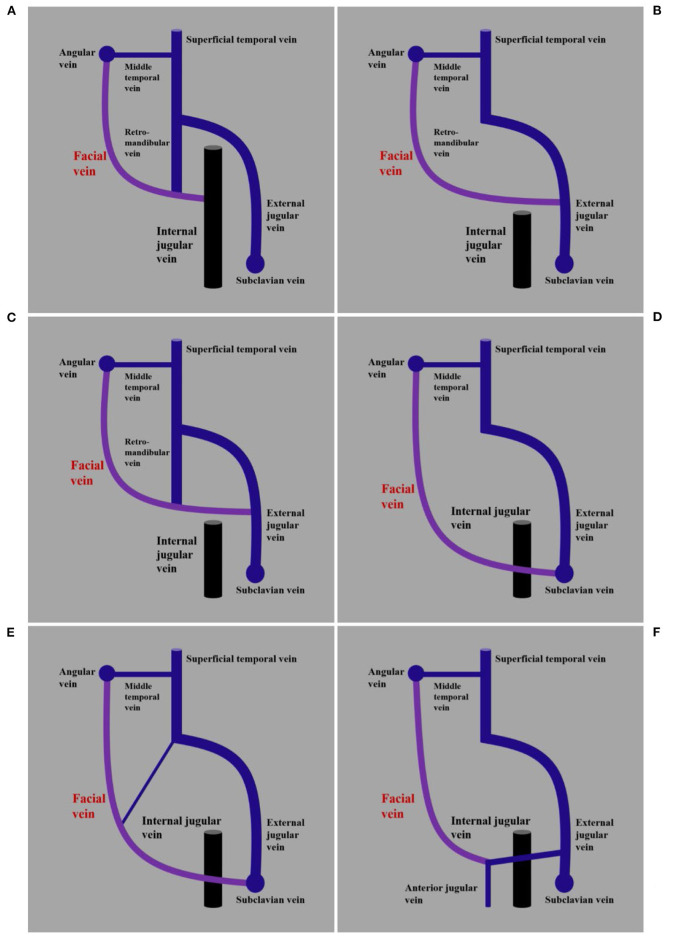
Illustrations of normal anatomy and variation of the FV. **(A)** Imaging shows the FV together with the retromandibular vein into the IJV. **(B)** Imaging shows the FV without the connection of the retromandibular vein into the EJV. **(C)** Imaging shows the FV without the connection of the retromandibular vein into the EJV. **(D)** Imaging shows the FV without the connection of the retromandibular vein into the subclavian vein. **(E)** Imaging shows the FV with the connection of the retromandibular vein into the subclavian vein. **(F)** Imaging shows the FV with the connection of other veins into the anterior jugular vein. AJV, anterior jugular vein; EJV, external jugular vein; FV, facial vein; IJV, internal jugular vein; MTV, middle temporal vein.

The normal anatomy and variation of the FV were summarized in the illustrations to provide a better understanding of the FV (Figure 4).

### 2.3. Venous valve

Previously, this FV system was often reported to be valveless. However, the valves in the inferior root of the SOV have been reported to prevent the flow from the FV and angular vein toward the SOV and the CS (12, 20). In addition, valves have also been reported in the FV (22). The valves set up obstacles for catheterization in the FV system.

## 3. TVE routes of CS-DAVF

The goal of EVT of the CS-DAVF is to occlude the retrograde drainage channel to the ophthalmic vein that has caused the ocular symptoms and to occlude the retrograde drainage channel to the cortical cerebral vein that might cause cerebral venous hypertension and hemorrhage (23). TVE allows thrombus formation in the CS to interrupt the fistulous communication of CS-DAVF (1). Many routes can be used to access CS-DAVFs to perform TVE, and these routes mainly include the IPS and SOV (1). In Meyers et al.'s (8) report of 117 TVEs of CS-DAVFs, access to the CS was achieved *via* the IPS or SOV in 76% of the cases. Other uncommon routes include the superior petrosal sinus, basilar plexus, superficial middle cerebral vein, sphenoparietal sinus, and pterygoid plexus (3, 7, 24, 25).

### 3.1. Trans-IPS or SOV routes with surgical exposure

In the great majority of CS-DAVFs, the trans-IPS route from the jugular bulb to the CS is the favorite and most direct route along the petrooccipital fissure (23). However, the trans-IPS route may fail when the IPS is stenosed or is hypoplastic/aplastic. Alternatively, there is no communication between the CS compartment involved in the DAVF and the IPS (26). At this time, if the SOV is patent and hyperplastic, an anterior approach through the SOV to the CS can be accessible through a surgical approach (20). The SOV is located in the superomedial quadrant of the orbit; in the surgical approach, it requires the cut-down or puncture of the SOV and may not be cosmetically acceptable (27, 28). Some disadvantages of surgical exposure include bleeding of the SOV and injury of the nerve and muscle (29).

### 3.2. Trans-FV-SOV route

Currently, improved techniques and materials allow SOV catheterization *via* a transfemoral trans-FV-SOV route (30). Complications from cut-down or puncture of the SOV can be avoided when using the transfemoral trans-FV-SOV route. However, the transfemoral trans-FV-SOV route is not always easily successful. In Kim et al.'s (3) report of the transfemoral trans-FV-SOV route to embolize CS-DAVFs, the technique's success was 13% (7/56) for CS-DAVFs. In a report by Klisch et al. (31), the technique's success rate was 50% (4/8 cases). In the Matsumoto et al.'s (4) literature review, the success rate ranged from 50 to 100%.

Except for the classical transfemoral trans-FV-SOV route to the CS, other similar paths have been reported. For instance, similar paths, such as the trans-retromandibular vein, trans-middle temporal vein, or trans-superficial temporal vein, and then the SOV to the CS can be used (23, 24, 32–35). Rarely, a path through the retromandibular vein, the pterygoid venous plexus, the deep FV, and the SOV to the CS when using this route was also reported (36). These routes were less frequently used and are not discussed in our review.

## 4. Procedure of transfemoral trans-FV-SOV route

Under general anesthesia and while fully heparinized (3,000 IU as a bolus and 1,000 IU/h as a continuous infusion to keep the activated clotting time to three to four times the normal rate), both transarterial and transvenous femoral or radial approaches are performed (23, 30, 37). Through the transarterial path, a diagnostic catheter is positioned in the carotid artery as the dominant feeding artery of the CS-DAVF to provide a forward venous roadmap (10).

**Figure 5:**
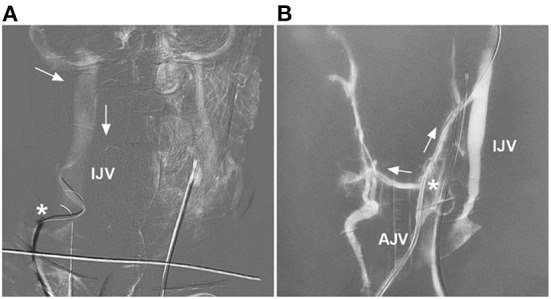
Forward and reverse venous roadmaps. **(A)** Forward roadmap: after the contrast medium was slowly injected into the carotid artery, the IJV was shown clearly to help the guiding catheter (asterisk) navigate in the IJV, and the arrows indicate the direction of blood flow. **(B)** Reverse roadmap: immediately after the guiding catheter (asterisk) injected the contrast medium into the AJV, the AJV and nearby veins were shown clearly, and the arrows indicate the direction of blood flow. AJV, anterior jugular vein; IJV, internal jugular vein.

Then, a transvenous guiding catheter is passed through the external iliac vein, the inferior vena cava, the right atrium, the superior vena cava, the brachiocephalic vein, the IJV or EJV or the subclavian vein, finally reaching or going into the orifice of the common FV at the mid-cervical region with the assistance of a forward venous roadmap from the draining vein of the CS-DAVF and a reverse venous roadmap in the vein from the guiding catheter (Figure 5) (38). After positioning a guiding catheter, following the microguidewire, retrograde catheterization of a microcatheter is performed through the FV, the angular vein, the SOV, and then into the CS under the assistance of forward and reverse venous roadmaps (39).

After the microcatheter reaches the fistula component, coiling is initiated to occlude the fistula. Liquid embolic materials, such as Onyx (Medtronic, Irvine, California, USA), can be used in combination to improve the tamponade effect of the coils and reduce the hemodynamic load on the petrosal–galenic system to avoid the escape of liquid embolic materials into the draining veins too far (20, 24, 40). The TVE endpoint was complete occlusion or slow flow of the CS-DAVF without dangerous cortical or deep venous drainage, and spontaneous thrombosis of the residual DAVF was expected (24).

**Figure 6:**
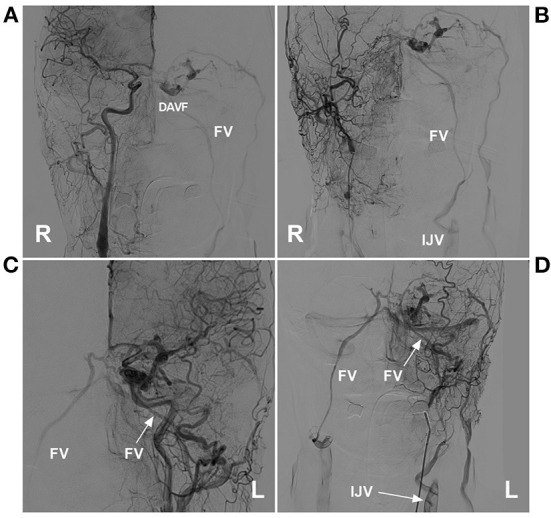
Preoperative angiography of CS-DAVF embolized *via* the trans-IJV-FV-SOV route. **(A, B)** Right ICA **(A)** and ECA **(B)** angiographies show the dural branches of the ICA and ECA supplied to the DAVF in the right CS. The left FV served as the draining vein into the IJV. **(C, D)** Left ICA **(C)** and ECA **(D)** angiographies show the dural branches of the ICA and ECA supplied to the DAVF. The bilateral FVs served as the draining veins. CS, cavernous sinus; DAVF, dural arteriovenous fistula; ECA, external carotid artery; FV, facial vein; ICA, internal carotid artery; IJV, internal jugular vein; L, left; R, right; SOV, superior ophthalmic vein.

**Figure 7:**
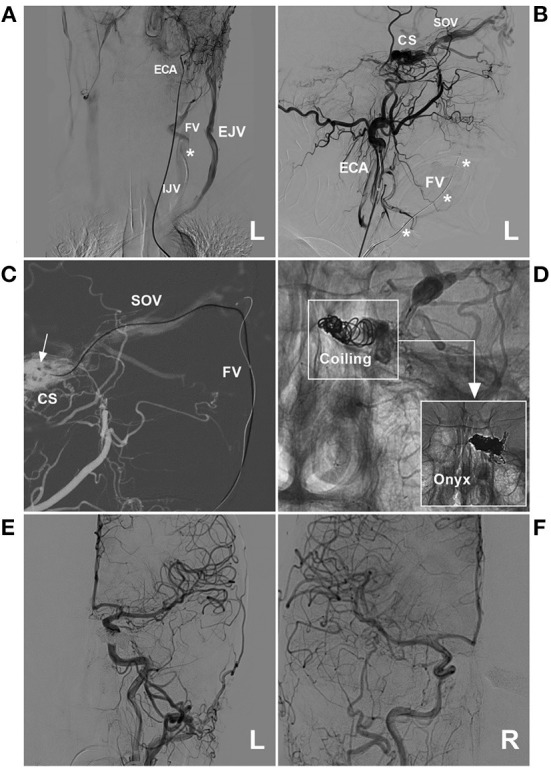
Operative angiography of CS-DAVF embolized *via* the trans-IJV-FV-SOV route. **(A)** Angiography of the left ECA *via* the transarterial diagnostic catheter showing that the transvenous therapeutic guiding catheter (asterisk) was placed into the IJV. **(B)** Angiography of the left ECA shows that the microcatheter was advanced into the FV (asterisks) *via* the transfemoral trans-IJV-FV route. **(C)** Venous navigation roadmap shows that the microcatheter passed through the angular vein and SOV into the CS (arrow). **(D)** Unsubtracted angiography showing coiling (frame) was performed; the picture in the picture (right angle arrow) shows the combined use of Onyx. **(E, F)** Post-TVE angiographies of the left carotid artery **(E)** and right carotid artery **(F)** show complete occlusion of the DAVF. CS, cavernous sinus; DAVF, dural arteriovenous fistula; ECA, external carotid artery; EJV, external jugular vein; FV, facial vein; IJV, internal jugular vein; L, left; R, right; SOV, superior ophthalmic vein; TVE, transvenous embolization.

**Figure 8:**
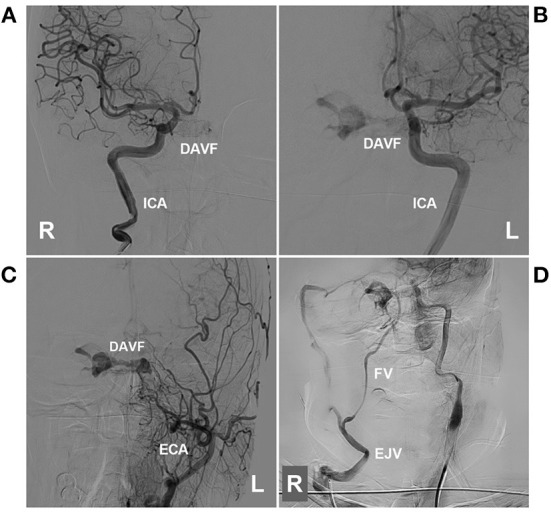
Preoperative angiography of CS-DAVF embolized *via* the trans-EJV-FV-SOV route. **(A, B)** Angiographies of right **(A)** and left **(B)** ICAs show the dural branches of the ICA supplied to the DAVF. **(C)** Angiography of the left ECA shows the dural branches of the ECA supplied to the DAVF. **(D)** Venous phase angiography of the right ICA shows that the right FV served as the draining vein of the DAVF into the EJV. CS, cavernous sinus; DAVF, dural arteriovenous fistula; ECA, external carotid artery; EJV, external jugular vein; FV, facial vein; ICA, internal carotid artery; L, left; R, right; SOV, superior ophthalmic vein.

**Figure 9:**
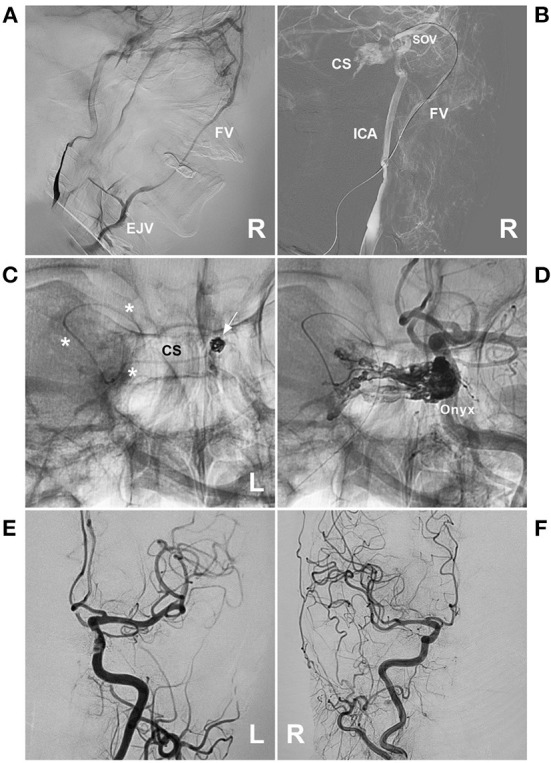
Operative angiography of CS-DAVF embolized *via* the trans-EJV-FV-SOV route. **(A)** Venous phase angiography of the right ICA shows the right FV into the EJV. **(B)** Venous navigation roadmap shows that the microcatheter passed through the right angular vein into the SOV. **(C)** X-ray image shows the microcatheter (asterisks) passing through the intercavernous sinus from the right into the left CS to perform coiling (arrow). **(D)** Unsubtracted angiography shows the combined use of Onyx to occlude the CS-DAVF. **(E, F)** Post-TVE angiographies of the left carotid artery **(E)** and right carotid artery **(F)** show complete occlusion of the DAVF. CS, cavernous sinus; DAVF, dural arteriovenous fistula; EJV, external jugular vein; FV, facial vein; ICA, internal carotid artery; L, left; R, right; SOV, superior ophthalmic vein; TVE, transvenous embolization.

Two typical cases of CS-DAVF treated with the transfemoral trans-FV-SOV route are provided to demonstrate the TVE procedure in Figures 6–9.

## 5. Difficulty and solution of transfemoral trans-FV-SOV EVT

**Figure 10:**
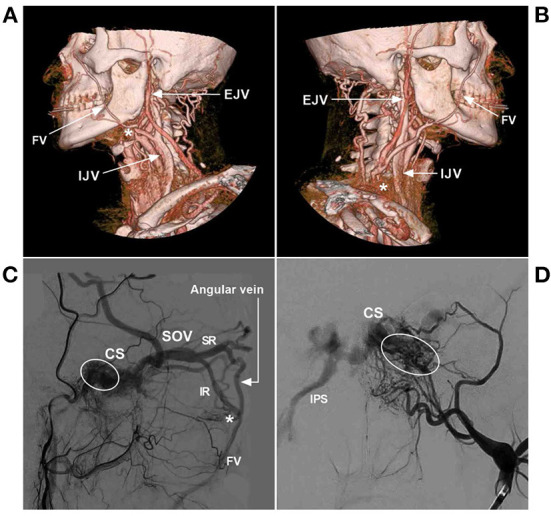
Difficulty of transfemoral trans-FV-SOV to embolize CS-DAVF. **(A)** Lateral view CTA shows the FV jointed into the IJV. The asterisk indicates the looping of the common FV, and catheterization was difficult. **(B)** Lateral view CTA shows the FV jointed into the EJV. The asterisk indicates the junction of the EJV and subclavian vein; here, venous valves exist. **(C)** Lateral view angiography of the ECA shows the veins of the orbit region. The asterisk indicates that venous valves exist at the inferior root of the SOV, and the circle indicates the fistula point of the CS-DAVF. **(D)** Anterior–posterior view angiography of the ECA shows the fistula point (circle) of the CS-DAVF. CS, cavernous sinus; DAVF, dural arteriovenous fistula; ECA, external carotid artery; EJV, external jugular vein; FV, facial vein; IJV, internal jugular vein; IPS, inferior petrous sinus; IR, inferior root; SOV, superior ophthalmic vein; SR, superior root.

Transfemoral trans-FV-SOV catheterization is often time-consuming, with erroneous attempts or technique failures. The difficulties include the catheterization of the junction of the IJV/EJV with the FV, the region of the orbital angular vein and SOV roots, and obliteration of the DAVF (Figure 10) (32).

### 5.1. Catheterization of the FV

When the FV originates from the IJV, some obstacles to catheterizing the FV include a hypoplastic FV or a very sharp and tortuous looping angle at the junction of the IVJ with the FV (Figure 10A). When the FV originates from the EJV, catheterization into the EJV usually takes more time because the EJV is smaller and lateral in location and has two pairs of valves that are present before its entrance into the subclavian vein (Figure 10B) (21).

In addition, the guiding catheter is often weak in the venous system because the vein lumen is larger than the arteries, and the wall tension is weaker than that in the arterial system (32). A transfemoral stiff 6F long sheath or 7F/8F thick guiding catheter to provide sufficient supporting force is helpful to overcome the obstacle to catheterizing the FV.

### 5.2. Catheterization of the angular vein and SOV

Another challenge is placing the microcatheter through the angular vein and tortuous SOV roots into the SOV. At the junction of the angular vein and the SOV, the vessels of the orbital venous system are often abruptly angled, stenosed, and tortuous and have numerous tiny branches (36). Sometimes, the SOV is angiographically occlusive due to early thrombosis (39). The SOV contains superior and inferior roots (Figure 10C) (15). Valves in the inferior root of the SOV may prevent the catheterization of the inferior root of the SOV (20).

Strong proximal support of the transfemoral catheter is crucial for the catheter to pass through the region. The FV was often large enough to cannulate it with 4F or even 5F intermediate catheters, which provided a solution for difficult catheterization (41, 42). At this time, a triple coaxial system by the telescopic method was helpful and feasible. First, a 6F long sheath, 7F/8F guiding catheter was placed in the IJV/EJV, and then a 4F/5F coaxial catheter was advanced into the angular vein or distal FV to strengthen the supporting force for a microcatheter into the SOV (20, 36, 41, 43, 44). Once the angular vein is cannulated with a microguidewire, the vessel course is straightened, which allows the microwire to pass the SOV (23). In a report by Fujita et al. (20), of the 10 CS-DAVFs that underwent TVE *via* transfemoral FV-SOV, the success rate of catheterization was 100% using a triple coaxial system.

However, in the triple coaxial system, the intermediate catheter should not be too long to guarantee that the microcatheter is long enough to be navigated into the CS. In addition, it was useful to combine both thick and thin microwires. After a thicker and stiffer microwire (such as 0.014 or 0.012 inches) was used to navigate the microcatheter to reach the angular vein, a thinner and softer microwire (such as 0.010 inches) was helpful to pass through the orbital venous system with looping or a double-angled microwire tip after the “J” configuration failure, and then the microcatheter could be placed into the SOV (32).

### 5.3. Determination of the fistula point and embolization of the DAVF

For TVE, it is crucial to confirm the fistula point, which presents with a tubular or elliptical structure that is separated from the CS, where multiple feeding arteries converge and continue to the CS (5). Selective angiographies of bilateral ICAs and ECAs are necessary. When the microcatheter is navigated into the CS, superselective venous angiography is performed to delineate the venous angioarchitecture of the DAVF (Figure 1B).

According to both arterial and venous angiographies, the location of the fistula point can be confirmed. The fistula point is often located at the posterior part of the CS (45). In the transfemoral trans-FV-SOV route to embolize CS-DAVF, coiling should target the posterior part of the CS first and then backward toward the SOV. When liquid embolic materials [such as Onyx et al. (46)] are used in combination, after coiling, the microcatheter should be advanced deeply into the middle of the mass of the coils to cast the liquid embolic material. Certainly, if the FV has a sufficient size to catheterize double microcatheters into the CS, the TVE was more convenient but also needed a sufficient supporting system (32, 47).

Certainly, there are other difficulties for the transfemoral trans-FV route to embolize CS-DAVFs. For instance, the slow flow of CS-DAVFs may make it difficult to create a good-quality roadmap to guide subsequent catheterization (10, 32).

## 6. Complications and prognosis

### 6.1. Complication

The transfemoral trans-FV-SOV route to embolize CS-DAVFs can be associated with two types of complications (3, 48). One type is procedure-related complications, such as the development of a vein perforating injury by the microwire or microcatheter, and the other type is embolization-related complications (Table 1).

**Table 1:** Complication of the trans-FV-SOV route to embolize CS-DAVF.

**Author/year**	**Type**	**Presentation**	**Cause**	**Resolution**
Halbach et al. (55)	Procedure-related	SOV hemorrhage	Injury or perforation by the microguidewire or microcatheter	Conservative treatment
Kim et al. (3)	Embolization-related	Brain stem congestion	Shunt of blood flow into pontomesencephalic vein	Repeated embolization
Luo et al. (21) and Choi et al. (32)	Embolization-related	Cranial nerve palsy	Mass effect and toxicity of embolic material	Conservative treatment

CS, cavernous sinus; DAVF, dural arteriovenous fistula; FV, facial vein, SOV, superior ophthalmic vein.

#### 6.1.1. Procedure-related complications

Unlike the IPS route, the trans-FV-SOV route is surrounded by soft tissue, and the vein wall is thinner and non-elastic, which carries a greater risk of procedural venous perforation of a tortuous FV, an angular vein or the SOV (20, 49). Venous rupture can cause bleeding and hematomas, which are generally not dangerous in the FV course but become more dangerous in the SOV, and bleeding can consequently compress the intraorbital structures (21, 50). To avoid injury, interventionists should gently manipulate the microdevices within the vein and observe the monitors very carefully (32). Fortunately, procedure-related complications are uncommon in trans-FV TVE for CS-DAVF (10, 15).

#### 6.1.2. Embolization-related complications

The embolization-related complications are the same as those of TVE *via* other transvenous approaches (20). For instance, when embolizing too many compartments of the CS, CS overpacking syndrome can occur, mainly presenting with cranial nerve palsies; therefore, the non-fistulous compartment of the CS should be preserved (3, 24). CS overpacking syndrome is often transient and relieved within a few days to weeks with conservative treatment (3, 20). During casting Onyx in CS, the trigemino-cardiac reflex can occur and result in reproducible bradycardia (51). The trans-FV-SOV route to embolize CS-DAVF may carry the risk of acute thrombosis of the SOV after the occlusion of the CS (30, 52, 53). Even central retinal vein thrombosis can occur, resulting in visual impairment (30). Therefore, heparin anticoagulation may be helpful.

Rarely, simple trapping or partial embolization of the involved compartment of the CS can lead to the diversion of shunt flow from the CS into the normal cerebral venous pathways and ultimately result in the conversion of CS-DAVF into a more dangerous disease, resulting in the development of cerebral infarct edema (54). In Kim et al.'s (3) report, two brain stem congestions by rerouting to the pontomesencephalic veins developed after the TVE of the CS-DAVF, which was a serious and rare complication, and repeated embolization may be needed.

### 6.2. The prognosis

For TVE for CS-DAVFs, the angiographic and clinical cure rates can reach 71–89 and 77–96%, respectively (3). Angiographic cure was defined as complete occlusion of the shunt or by a nearly complete occlusion in a small residual stagnant shunt that is considered likely to thrombose. These are considered successful angiographic results, and clinical cure was defined as the resolution of the symptoms related to the lesion (3).

For the transfemoral trans-FV-SOV route to embolize CS-DAVFs, good outcomes can be obtained. For instance, in Kim et al.'s (10) report of the trans-IJV-FV-SOV route for 12 CS-DAVFs and in Bionde et al.'s (15) report of the trans-IJV-FV-SOV route for seven CS-DAVFs, the symptoms and signs related to CS-DAVF all gradually resolved. In addition, in a report by Fujita et al. (20), 10 CS-DAVFs were treated with the trans-FV-SOV route, 32 CS-DAVFs were treated with the IPS route, and there were no differences in the neurological outcomes, which indicated that the trans-FV-SOV route to embolize CS-DAVFs was feasible and safe in selective cases.

## 7. Summary

Based on the review and our experience, we found that in the treatment of CS-DAVF, the transfemoral trans-FV-SOV route provides a valuable alternative to other transvenous routes and can be performed in selective cases. However, the route to embolize CS-DAVFs is often challenging due to considerable variations in the head and neck veins. A good prognosis with few complications can be obtained, similar to other transvenous routes.

## Author contributions

JY contributed to the conception and design of the review. ZZ collected the data. JY and ZZ contributed to drafting the text and preparing the figures. JY, ZZ, and KX revised the manuscript. All authors read and approved the final manuscript.
